# Core module biomarker identification with network exploration for breast cancer metastasis

**DOI:** 10.1186/1471-2105-13-12

**Published:** 2012-01-18

**Authors:** Ruoting Yang, Bernie J Daigle, Linda R Petzold, Francis J Doyle

**Affiliations:** 1 Institute for Collaborative Biotechnologies, University of California Santa Barbara, Santa Barbara, CA 93106-5080, USA; 2 Department of Computer Science, University of California Santa Barbara, Santa Barbara, CA 93106-5110, USA; 3 Department of Mechanical Engineering, University of California Santa Barbara, Santa Barbara, CA 93106-5070, USA; 4 Department of Chemical Engineering, University of California Santa Barbara, Santa Barbara, CA 93106-5080, USA

## Abstract

**Background:**

In a complex disease, the expression of many genes can be significantly altered, leading to the appearance of a differentially expressed "disease module". Some of these genes directly correspond to the disease phenotype, (i.e. "driver" genes), while others represent closely-related first-degree neighbours in gene interaction space. The remaining genes consist of further removed "passenger" genes, which are often not directly related to the original cause of the disease. For prognostic and diagnostic purposes, it is crucial to be able to separate the group of "driver" genes and their first-degree neighbours, (i.e. "core module") from the general "disease module".

**Results:**

We have developed COMBINER: COre Module Biomarker Identification with Network ExploRation. COMBINER is a novel pathway-based approach for selecting highly reproducible discriminative biomarkers. We applied COMBINER to three benchmark breast cancer datasets for identifying prognostic biomarkers. COMBINER-derived biomarkers exhibited 10-fold higher reproducibility than other methods, with up to 30-fold greater enrichment for known cancer-related genes, and 4-fold enrichment for known breast cancer susceptible genes. More than 50% and 40% of the resulting biomarkers were cancer and breast cancer specific, respectively. The identified modules were overlaid onto a map of intracellular pathways that comprehensively highlighted the hallmarks of cancer. Furthermore, we constructed a global regulatory network intertwining several functional clusters and uncovered 13 confident "driver" genes of breast cancer metastasis.

**Conclusions:**

COMBINER can efficiently and robustly identify disease core module genes and construct their associated regulatory network. In the same way, it is potentially applicable in the characterization of any disease that can be probed with microarrays.

## Background

In recent years, gene expression signatures based on DNA microarray technology have proven useful for predicting the risk of breast cancer. Agendia's MammaPrint has become the first FDA-cleared breast cancer prognosis marker chip containing 70 gene signatures [1]. Many other microarray-based biomarkers, such as 76 gene signatures [2] have been derived using independent data sources. However, there are only three overlaps between MammaPrint's 70-gene and Wang's 76-gene signatures. Furthermore, many of these markers are functionally unrelated to breast cancer. In order to identify robust, functionally relevant disease biomarkers, it is crucial to find gene signatures that are consistent in various data sources.

A complex disease such as breast cancer results in many differentially expressed genes (DEGs), which together can be used to construct a "disease module" network [3]. Some of these DEGs directly correspond to the disease phenotype (i.e. "driver" genes). The expression changes enacted on the driver genes lead to a cascade of changes of other genes: initially to their first-degree interaction neighbors [4], followed by downstream effects to so-called "passenger" genes. Due to their direct relevance to the biology of the disease in question, the expression changes of the driver genes and their first-degree neighbours (i.e. members of the "core module"), should be more consistent than those of the passenger genes when compared across independent cohorts. However, it is often difficult to separate the core module from the passenger genes for a given disease [5,6]. In this paper, we aim to isolate the core module from the more general disease module and further identify the driver genes using network analysis.

The most intuitive way of finding the disease core module is to identify the Differential Expressed Genes (DEGs) over various cohorts. Unfortunately, the typically larger number of passenger genes in each cohort will contribute to the majority of gene overlaps, due to statistical chance. A more biologically-motivated technique for identifying the core module is to find overlapping differentially expressed pathways. However, a pathway may also contain hundreds of genes with respect to the disease in question, while only a functional submodule (a small group of genes) is differentially expressed. These submodules are often overlooked in pathway enrichment analysis.

In light of the aforementioned challenges, we propose to identify Pathway Activities (PAs) from cohorts of data and use supervised classification to isolate a consistent core module. Each PA is a vector aggregating the information of a few genes expressed in a pathway [7,8]. The use of PAs for biomarker identification has been shown improve reproducibility and disease-related functional enrichment of the resulting biomarkers [7]. The main idea behind our method is to infer the most significant PAs in each data cohort, and validate these PAs using classification methods in other cohorts. If a PA also scores highly in all the other cohorts, we consider it to be consistently differentially expressed in the disease of interest. Furthermore, we would consider the genes that make up the PA to belong to the disease core module.

**Figure 1:**
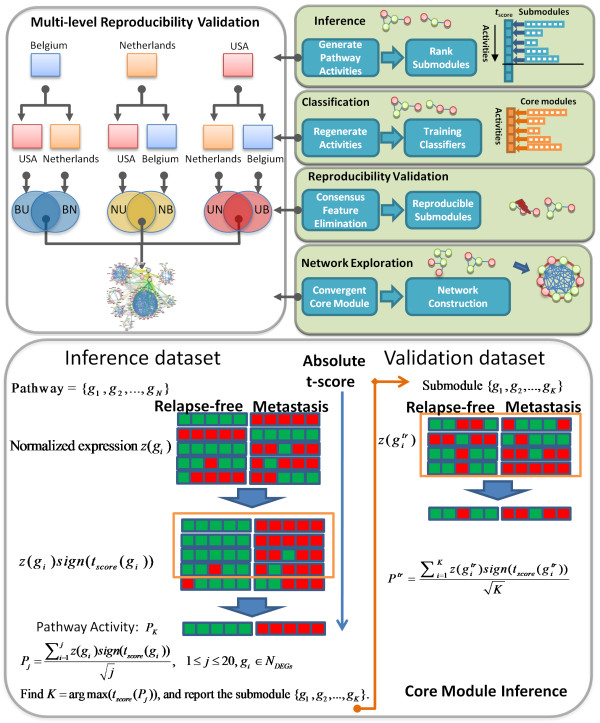
**Schematic overview of COMBINER**. COMBINER uses Core Module Inference (CMI) to infer candidate pathway activities from each pathway in an inference dataset, Consensus Feature Elimination (CFE) to filter out irreproducible activities in validation datasets, and a multi-level reproducibility validation framework to conduct pair-wise validations to find common reproducible activities which make up the "core module". To identify the driver genes, we reassemble the resulting core module markers in both intracellular signalling pathways and a large overall regulatory network reflecting interactions between pathways.

In this work, we develop a novel biomarker identification framework entitled COre Module Biomarker Identification with Network ExploRation (COMBINER). COMBINER identifies "core module" (Figure 1) that are consistently differentially expressed as a whole in the data cohorts of interest. COMBINER uses a Core Module Inference (CMI) component to infer candidate PAs from pathway database, a Consensus Feature Elimination (CFE) component to filter out irreproducible PAs, and a multi-level reproducibility validation framework to find the consistent PAs, which in turn make up the complete core module. In its final step, COMBINER uses known pathways and protein networks to identify the driver genes within this core module.

To illustrate its utility, we apply COMBINER to three benchmark breast cancer datasets. We evaluate the resulting core module for accuracy, reproducibility, and enrichment for known cancer-related genes. We then explore the roles of the COMBINER-identified core module in the hallmarks of cancer, and we reconstruct a breast cancer-specific interaction network composed of functionally coherent modules. Finally, we summarize our analyses by identifying 13 high confidence driver genes from COMBINER markers.

## Results and Discussion

### Overview

COMBINER is a multi-level optimization framework for identifying core module markers (Figure 1 and Methods). Briefly, COMBINER infers candidate submodules from known pathways, identifies the reproducible "core module" using independent cohorts, and uses intracellular signaling pathways and protein networks to identify the "driver" genes from the "core module".

We applied COMBINER to three independent breast cancer datasets to evaluate its effectiveness: Netherlands [9], USA [2], and Belgium [10]. We obtained pathway information from the MsigDB v3.0 Canonical Pathways subset [11]. To decrease redundancy, we applied pathway filtering to remove bulky pathways such as KEGG Pathways of Cancer. This resulted in a pathway dataset containing 624 pathways with 5,155 genes assayed in all three benchmark datasets.

### Core Module Inference improves reproducibility and classification accuracy

**Figure 2:**
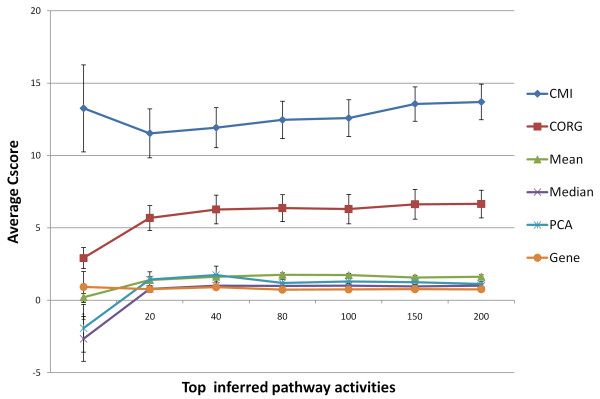
**Reproducible power of pathway inference methods**. The reproducibility power of a pathway inference method in an inference-validation pair datasets is measured by $C_{score}\left(N\right)=\frac{1}{N}\sum_{i=1}^{N}t_{score}\left(P_{I}^{i}\right)\cdot t_{score}\left(P_{V}^{i}\right)$, where $P_{I}^{i}$ is the *i*^th ^PA in descending order in the inference dataset, $P_{V}^{i}$ is its corresponding PA in the validation dataset, and *N *is the number of selected inferred pathways. The overall reproducibility is then defined as the average Cscore of selected top inferred pathway activities over all six inference-validation pairs. We compared CMI with five inference methods, including the CORG, mean, median, first component score of PCA, as well as no-inferring gene method. Comparing by different ranges of top inferred activities, the CMI showed significant better overall reproducibility over other methods.

A primary challenge of pathway inference is to find pathway subsets that are reproducible between independent datasets. We compared Core Module Inference (CMI) with five other inference methods as well as individual genes (see Methods). When compared to a range of numbers of inferred Pathway Activities (PAs), CMI showed two-fold increased reproducibility over the related CORG method and about a 10-fold improvement over other methods (Figure 2).

**Figure 3:**
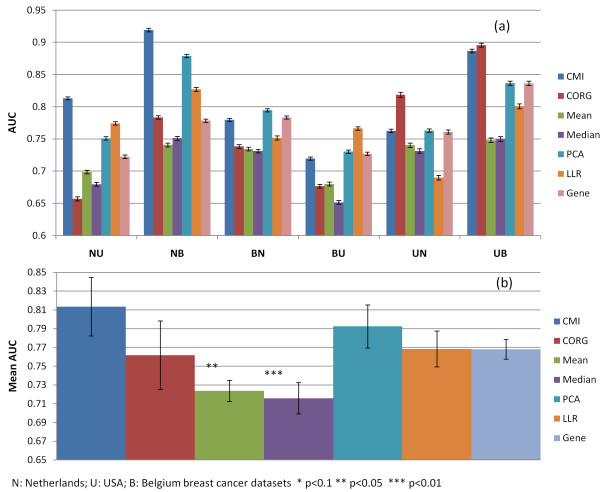
**Comparison of CMI and other inference methods-based COMBINER using LDA-CFE classifiers focused on the top 100 inferred pathways**. Seven methods were compared here, including CMI, CORG, Mean, Median, PCA, LLR and Individual Gene. (a) Classification accuracy for best feature set: pair-wise comparisons. Starting from all 100 inferred pathway activities, we recursively removed the activity with the lowest average weight from 500 LDA classifiers, until the maximum average AUC was reached. The process was repeated 100 times and the most frequently occurring marker set was regarded as the ultimate marker. We measured classification accuracy of each method by computing AUC mean ± standard error for the final feature set. (b) Classification accuracy overall. The overall classification accuracy was measured by computing the average maximum mean AUC of all six inference-validation pairs. On average, CMI was superior to the other methods, even though its activity vector consisted of expression values from only a few genes in each pathway.

We then compared the classification accuracy of CMI and the other inference methods using Linear Discriminant Analysis-Consensus Feature Elimination (LDA-CFE) classifiers focused on the top 100 inferred PAs (Methods). As shown in Figure 3, COMBINER run using PA vectors identified by CMI (CMI-COMBINER) exhibits better overall accuracy than the other methods coupled with COMBINER. Similarly, CMI also shows good overall accuracy using the SVM classifier (Additional file 1, Figure S1).

### Core module markers enrich cancer-related genes

We compared the enrichment of known cancer genes in the biomarkers discovered by CMI-COMBINER, (93 genes); CORG-COMBINER, (i.e. COMBINER run using CORG activity vectors), (123 genes); Subnetwork markers (1162 genes) ( [7], http://www.cellcircuits.com); MammaPrint's 70-gene signature (G70) (70 genes) [1]; and Wang's 76-gene signature (G76) (76 genes) [2]. Seven known cancer gene datasets were compared (see Materials and methods). Both CMI-COMBINER and CORG-COMBINER showed much higher enrichment of cancer-related genes in their biomarker signatures (Table 1). Specifically, CMI- and CORG-COMBINER showed up to 4-fold increased enrichment over subnetwork markers and up to 30-fold enrichment over other gene signatures. In particular for known breast cancer genes in Census, they exhibited up to 4 fold enrichment over others. More than 50% and 40% of the resulting biomarkers are cancer and breast cancer specific, respectively. Additionally, CMI-COMBINER showed greater enrichment than CORG-COMBINER with respect to the Atlas of Cancer Genes, which is the largest cancer gene collection. Consistent to Chuang et al's results [7],. we also found insignificant enrichment in CANgene dataset including 122 mutative genes from 11 breast cancer cell lines. A possible explanation is that "the cancer cell lines capture a different disease state than that found in the population of patients surveyed by microarray profiling." [7] The COMBINER core module markers with associated pathways are summarized in Additional file 2, Table S1 and Additional file 3, Table S2. Additional file 4, Table S3 lists the overlaps between CMI-/CORG-COMBINER and KEGG pathways of cancer, along with up-/down-regulation information.

**Table 1:** Cancer Gene Enrichment rate of various breast cancer gene signatures

	CMI-COMBINER	CORG-COMBINER	Subnetwork	G70	G76
NetPath	54.17%*	50.41%*	26.33%*	10.00%	10.53%
Atlas	60.42%*	46.34%	32.87%	15.71%	18.42%
Census	11.46%*	13.82%*	5.42%*	2.86%	0.00%
CANgene	1.04%	1.63%	0.52%	0.00%	0.00%
G2SBC	43.75%*	46.34%*	19.02%	21.43%	10.53%
COSMIC	16.67%	17.89%*	7.06%	4.29%	1.32%
KEGG	35.42%*	29.27%*	9.90%*	8.57%	1.32%

* p-value < 0.05 for hypergeometric tests

### Core module markers highlight the hallmarks of cancer

**Figure 4:**
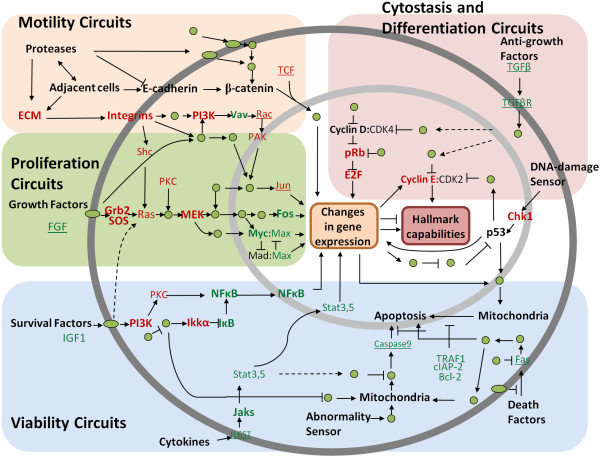
**COMBINER biomarkers overlap with well-known cancer-related signalling pathways**. The core module markers from CMI and CORG are listed in normal and italic fonts, respectively, while the common markers are in bold. Red/green color denotes up-/down-regulation. The remaining proteins in the circuit are abstracted as unlabeled nodes. The common core module markers of CMI- and CORG-COMBINER describe growth factors, survival factors, the cell cycle, and the extracellular matrix. Unique pathways to CMI-COMBINER include the anti-apoptosis and JAK-STAT cascade, while anti-growth factor and death factor pathways were discovered uniquely by CORG-COMBINER.

As shown in Figure 4, the COMBINER-discovered biomarkers are overlaid on the hallmarks of cancer [12,13], which integrate the common intracellular signalling pathways of all subtypes of cancer. The components of the core module markers from CMI and CORG along with eighteen common markers are listed in different fonts. The remaining proteins (most were not differentially expressed) in the pathways are consolidated into unlabeled nodes. Figure 4 shows that the identified core module genes comprehensively highlight the hallmarks, demonstrating the high specificity of COMBINER. In particular, 18 common markers, which we regard as the most reliable predictors, describe well-characterized processes involving growth factors, survival factors, the cell cycle, and the ExtraCellular Matrix (ECM). The modules unique to CMI-COMBINER include anti-apoptosis and JAK-STAT cascades, while pathways describing anti-growth factors and death factors were unique to CORG-COMBINER. A few well-known mutant proteins, including cyclin D1 and p53, may play an important role in connecting other signatures [7], but they showed only limited predictive ability in the three breast cancer datasets.

### Core module markers in predicted protein-protein interaction networks underpin functional modules

**Figure 5:**
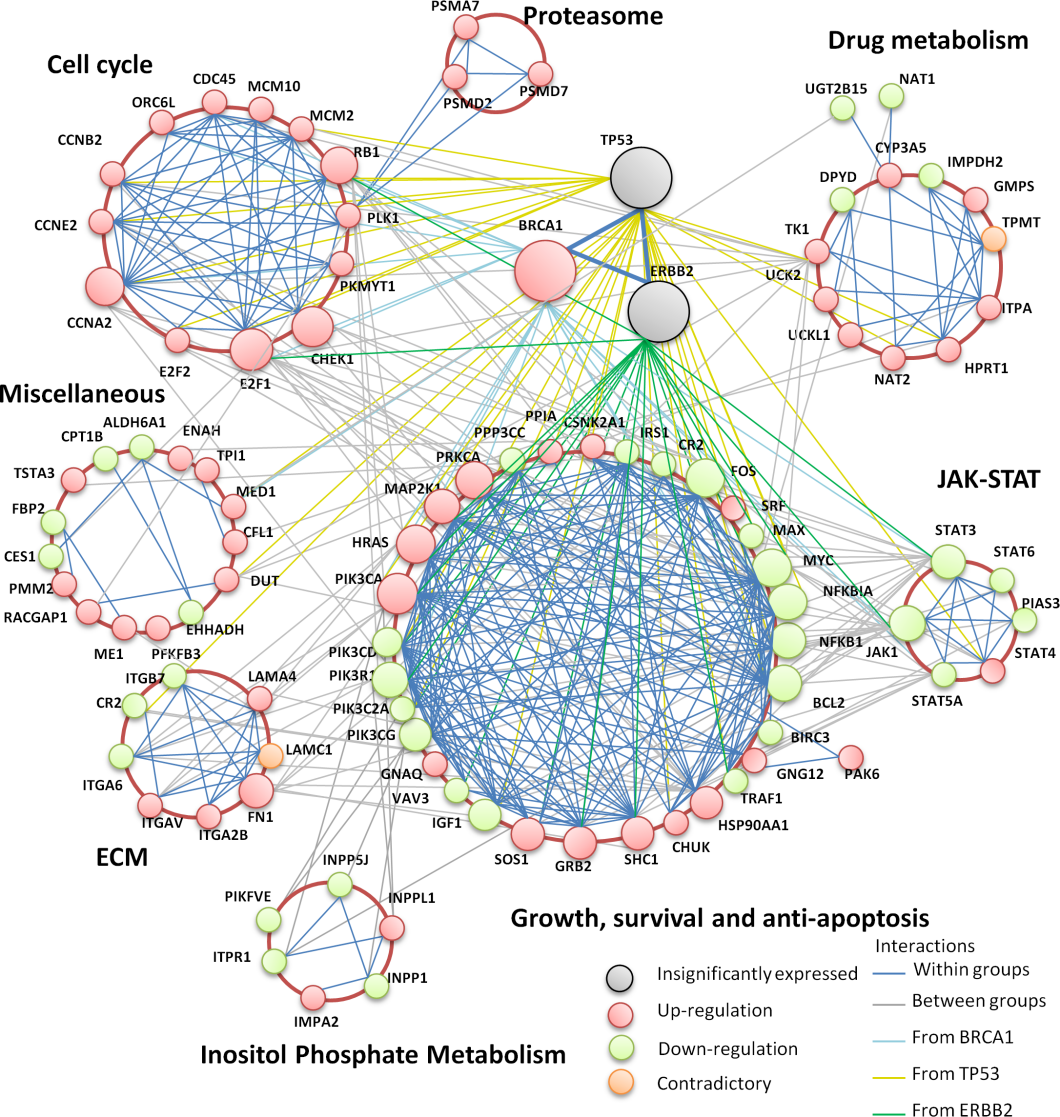
**Regulatory networks of CMI-COMBINER biomarkers The pink/green nodes denote up-/down-regulation of gene expression**. The orange nodes indicate contradictory regulation in different datasets. Larger nodes are highly connected in the network; most are overlaps between CMI- and CORG-COMBINER. The three well-known oncogenes for breast cancer metastasis-TP53, BRCA1, and ERBB2-were enlarged further. The core module markers were reassembled into an overall interaction network. Known functional modules neatly overlay well-connected clusters. Many of the highly connected genes are known "driver" genes playing an important role in breast cancer metastasis.

Figure 5 shows how a regulatory network was constructed using the interactome of the core module markers. The regulatory network was divided into a few functional modules, including cell cycle and ECM. These functional modules were interconnected by 20 "hub" genes (larger pink/green nodes), 13 of which overlapped with the common marker genes (Additional file 2, Table S1). Our results imply that these 13 "hub" markers are the essential "driver" genes of breast cancer metastasis (Table 2). For example, BRCA1 is among the most well-characterized genes whose mutation gives rise to breast cancer. In addition, low E2F1 transcript levels strongly predicted good prognosis based on quantitative RT-PCR in 317 primary breast cancer patients [14]. We further enlarged the nodes of three standard breast cancer indicators TP53, BRCA1, and ERBB2, which connect many of the surrounding hub genes. Although TP53 and ERBB2 are useful for a mechanistic understanding of breast cancer, they were not identified as discriminative gene markers. A regulatory network was also created representing CORG-COMBINER (Additional file 5, Figure S2), but no additional "hub" markers were found.

**Table 2:** Confident "driver" genes for breast cancer metastasis

Symbol	Entrez	Description
MAP2K1 [32]	5604	mitogen-activated protein kinase kinase 1
E2F1 [14]	1869	E2F transcription factor 1
GRB2 [33]	2885	growth factor receptor-bound protein 2
NFKB1 [34]	4790	nuclear factor of kappa light polypeptide gene enhancer in B-cells 1
RB1 [35]	5925	retinoblastoma 1
BRCA1 [36]	672	breast cancer 1, early onset
FOS [37]	2353	v-fos FBJ murine osteosarcoma viral oncogene homolog
SOS1 [38]	6654	son of sevenless homolog 1 (Drosophila)
PIK3CA [39]	5290	phosphoinositide-3-kinase, catalytic, alpha polypeptide
JAK1 [40]	3716	Janus kinase 1
SHC1 [41]	6464	SHC (Src homology 2 domain containing) transforming protein 1
MYC [42]	4609	v-myc myelocytomatosis viral oncogene homolog (avian)
CCNA2 [37]	890	cyclin A2

## Conclusions

Identifying accurate and reproducible disease biomarkers is an important challenge for gene expression analysis. To facilitate this task, we developed COMBINER, a novel pathway-based biomarker identification method that extracts the essential "core module" of disease from known biological networks. Compared to existing methods, COMBINER substantially improves the reproducibility and cancer-specific enrichment of its resulting biomarkers. We examined the identified markers in intracellular signalling networks highlighting the hallmarks of cancer. Reassembling the core module genes into a regulatory network, we found 13 "driver" genes connecting eight functional modules. We anticipate such molecular descriptions to prove even more useful when applied to diseases that are less well-characterized; our current work focuses on several such applications.

## Methods

### Gene expression, pathways, cancer gene databases, and interactome

We used three breast cancer datasets from different countries of origin to evaluate our method: Netherlands [9], USA [2], and Belgium [10]. Each dataset recorded whether the assayed patients developed metastasis within 5 years after surgery. The Netherlands, USA, and Belgium datasets contain expression profiles for 295, 286, and 198 patients, respectively, with 78, 107, and 35 patients experiencing metastasis. All of the patients in the USA and Belgium datasets had lymph-node-negative disease, although their estrogen receptor (ER) types differed. The Netherlands data contained both lymph-node positive and negative disease patients with differing ER types, 130 of which received adjuvant systemic therapy including chemotherapy and hormonal therapy. We performed a two-tailed t-test on the gene expression values of each dataset to distinguish between metastatic and non-metastatic patients, considering genes with p-value ≤.05 as differentially expressed (DE).

The reference cancer genes for enrichment analysis were collected from datasets including NetPath [15] (all cancers, http://www.netpath.org/), Atlas of Cancer Genes [16] (all cancers, http://atlasgeneticsoncology.org/), Census Genes [17] (all cancers), CANgenes [18] (breast cancer), G2SBC [19] (breast cancer, http://www.itb.cnr.it/breastcancer/), and KEGG Pathways of Cancer [20] (all cancers, KEGG hsa05200 http://www.genome.jp/kegg/pathway/hsa/hsa05200.html).

Pathway information was obtained from the MsigDB v3.0 Canonical Pathways subset [11,21]. This collection contains 880 pathways collected from seven hand-curated pathway databases including KEGG, Reactome, and Biocarta.

Predicted protein protein interaction information was obtained from STRING 9 [22].

### Core Module Inference

The CMI method adopts the strategy of the CORG method [8] of finding the genes with the most discriminative power, differing in three ways: first, the CORG method collects CORGs only from the up- or downregulated subset of genes in a pathway, and some key genes can thus be discarded. In contrast, CMI considers both up- and downregulation together. Second, CMI improves the greedy search for the discriminative set of genes. Third, CMI considers only differentially expressed genes. As illustrated in Figure 1, given a pathway consisting of genes {*g*_1_,... *g*_*i*_, ..., *g*_*n*_} ranking by a descending order of their absolute t-scores, with their normalized expression values {*z*(*g*_1_),..., *z*(*g*_*n*_)}, determining a core module {*g*_1_,..., *g*_*K*_} is equivalent to finding the *K*^th ^component, such that

(1) $$K=\text{arg}\text{max}\left(t_{score}\left(P_{j}\right)\right),$$

where

(2) $$P_{j}=\{\begin{array}{cc} \frac{{\sum^{\text{​}}}_{i=1}^{j}z(g_{i})sign(t_{score}(g_{i}))}{\sqrt{j}},1\leq j\leq min(|g_{i}\in DEGs|,20), & |g_{i}\in DEG_{s}|>0,\\ 0\;\;\;\;\;\;\;\;\;\;\;\;\;\;\;\;\;\;\;\;\;\;\;\;\;\;\;\;\;\;\;\;\;\;\;\;\;\;\;\;\;\;\;\;\;\;\;\;\;\;\;\;\;\;\;\;\;\;\;\;\;\;\;\;\;\;\;\;\;\;\;\;\;\;\;\;\;\;\;\;\;\;\;\;\;\;\;\;\;\;\;\;\;, & |g_{i}\in DEG_{s}|=0. \end{array}$$

*g*_*i *_is the *i*^th ^DEG in descending order and *Pj *is the PA containing from *g*_1 _to *g*_*j*_. | *g*_*i *_∈ *DEGs *| denotes number of DEGs in the pathway. The DEGs by default are the genes with p-value ≤ 0.05 in a two-tailed t-test. We limit the largest marker size to 20 DEGs. In fact, all marker sets have fewer than 20 components.

### Reproducibility power

We consider an inference-validation pair datasets to be reproducible if their pathway activities provide similar discriminative power. First, we rank the PAs inferred from the inference dataset in descending order by their tscores. Then, we define reproducibility by

(3) $$C_{score}\left(N\right)=\frac{1}{N}\sum_{i=1}^{N}t_{score}\left(P_{I}^{i}\right)\cdot t_{score}\left(P_{V}^{i}\right),$$

where $P_{I}^{i}$ is the *i*^th ^PA in descending order in the inference dataset, and $P_{V}^{i}$ is its corresponding PA in the validation dataset. For the breast cancer datasets, the overall reproducibility is then given by the average Cscore of the inferred pathways over all six inference-validation pairs.

Six methods were compared in this work, including CMI, CORG [8], Mean [23], Median [23], PCA [24], and Individual Gene. LLR(Log likelihood Ratio, [25]) was not compared here, because it is not discussed in the same gene expression space.

### Consensus Feature Elimination (CFE)

In this work, gene expression and activity vectors are generalized as features for classification. Given a set of features {***x ***_1_, ***x***_2_,..., ***x***_*n*_} with class labels {*y*_1_, *y*_2_,..., *y*_*n*_} ∈ {-1, +1}, the task of binary classification is to find a decision function

(4) $$D\left(x\right)\left\{\begin{array}{c} >0\Rightarrow x\in class\left(+\right)\\ <0\Rightarrow x\in class\left(-\right)\\ =0\Rightarrow x\in decisionboundary, \end{array}\right)$$

We choose a linear decision function, which can be described as a separating hyperplane:

(5) D(x)=w⋅x+b,

with ***w ***the weight vector and ***b ***the bias value.

**Figure 6:**
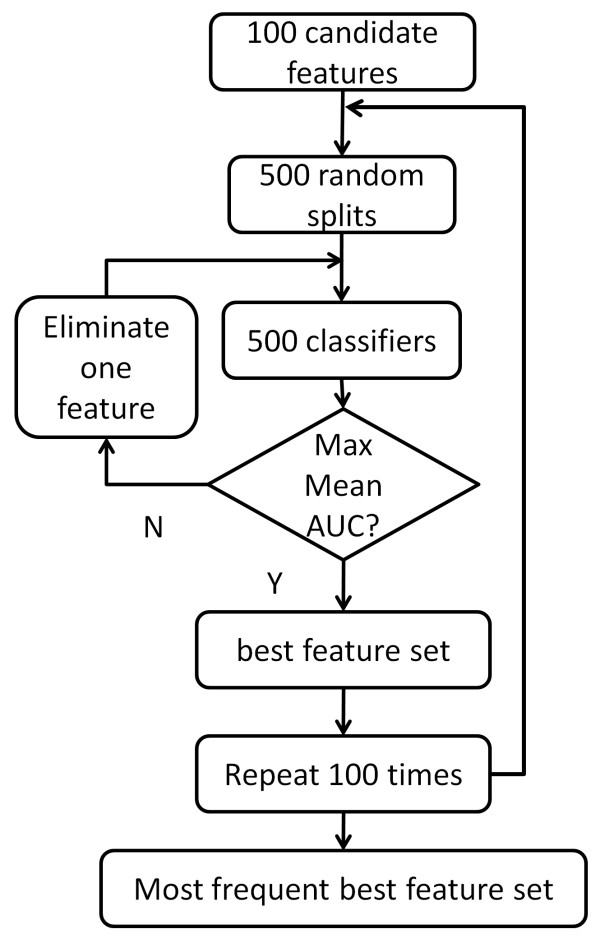
**Diagram of Consensus Feature Elimination**. We first generated 100 alternative 5-fold random splits of samples, upon which it constructs 500 classifiers with their AUCs as well as weight vectors. Each feature is then ranked by its average square weight. The lowest ranking feature was removed backward until the maximum average AUC was achieved. The procedure is repeated for 100 times, and the most frequently occurring marker set was regarded to be the ultimate marker.

Linear classifiers such as Linear Discriminant Analysis (LDA) [26] and linear Support Vector Machines (SVM) [27] use differing optimization criteria to estimate the weight vector. Intuitively, the weights indicate the importance of the associated features. Guyon *et al *proposed Recursive Feature Elimination (RFE), which removes features recursively based on their weights [28]. However, classical RFE exhibits lack of stability in feature selection [29]. In contrast to binary classification tasks that emphasize maximization of classification accuracy, biomarker identification requires features that are both accurate and reproducible across multiple experiments. Thus, we propose a Consensus Feature Elimination (CFE) approach to improve the stability of RFE. As illustrated in Figure 6, we first generate 100 alternative 5-fold random splits of samples, upon which we construct 500 classifiers and record their AUCs (Area Under Receiver Operating Characteristic Curves) and weight vectors. Each feature was then ranked by average square weight $\bar{w}=\sum_{j=1}^{500}{\left(w^{j}\right)}^{2}/500$. The lowest ranking feature was removed recursively until the maximum average AUC was achieved. This process, which has also been called Multiple RFE [30] or ensemble feature selection [31] is known to increase biomarker reproducibility and accuracy by as much as 30% and 15%, respectively. For the breast cancer datasets described in this work, we found the maximum AUC to be very stable, while the corresponding biomarker set was not always unique. Thus we chose to repeat the above procedure 100 times, selecting the most frequently occurring biomarkers as the final marker set.

Seven methods were compared in this work, including CMI, CORG [8], Mean [23], Median [23], PCA [24], LLR [25], and Individual Gene.

### Cancer gene enrichment analysis

The cancer gene enrichment analysis examines over-representation of known cancer genes in a gene signature. Assuming the total number of genes *N*, cancer genes *M*, and signature genes *J*, the probability of having more than *K *cancer genes in a signature follows a hypergeometric distribution:

(6) $$P(\#\;\text{of cancer genes}>K)=1-\sum_{i=0}^{K}\frac{\left({}_{i}^{J}\right)\left({}_{M-i}^{N-J}\right)}{\left({}_{M}^{N}\right)}.$$

### Software

COMBINER was implemented in Matlab R2010a with Bioinformatics toolbox v3.5. The source code is available on http://www.ruotingyang.com.

## Authors' contributions

RY, BJD, LRP, and FJD conceived and designed the research. RY, and BJD performed the analysis, the statistical computations, and wrote the paper. RY implemented the programs. All authors read and approved the final manuscript.

## Supplementary Material

Additional file 1 **Figure S1: Comparison of CMI and other pathway inference methods using SVM-CFE classifiers subject to top 100 inferred pathways**. Click here for file

Additional file 2 **Table S1: List of core module genes identified by CMI and CORG**. Click here for file

Additional file 3 **Table S2: Pathway markers identified by all methods**. Click here for file

Additional file 4 **Table S3: List of core module genes overlaid in KEGG pathway of cancers**. Click here for file

Additional file 5 **Figure S2: Unique core module of cancer pathway identified by CORG-COMBINER method**. Click here for file
