# Supplementary figures and images for: Core module biomarker identification with network exploration for breast cancer metastasis
Source: BMC Bioinformatics. 2012 Jan 18;13:12. doi: 10.1186/1471-2105-13-12 (PMC3349569; doi:10.1186/1471-2105-13-12)

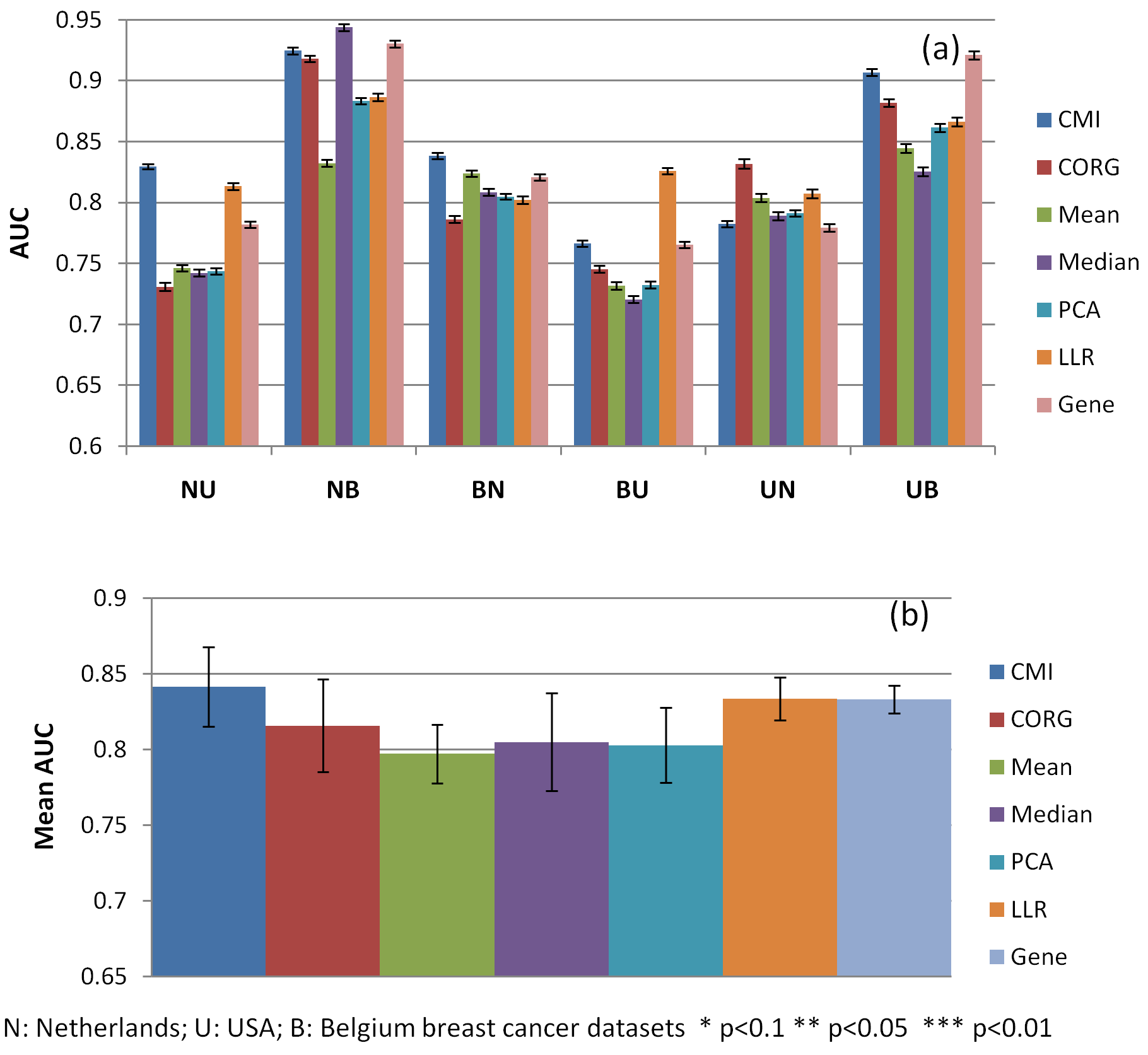

Supplement: Additional file 1 — Figure S1: Comparison of CMI and other pathway inference methods using SVM-CFE classifiers subject to top 100 inferred pathways. [file 1471-2105-13-12-S1.TIFF]

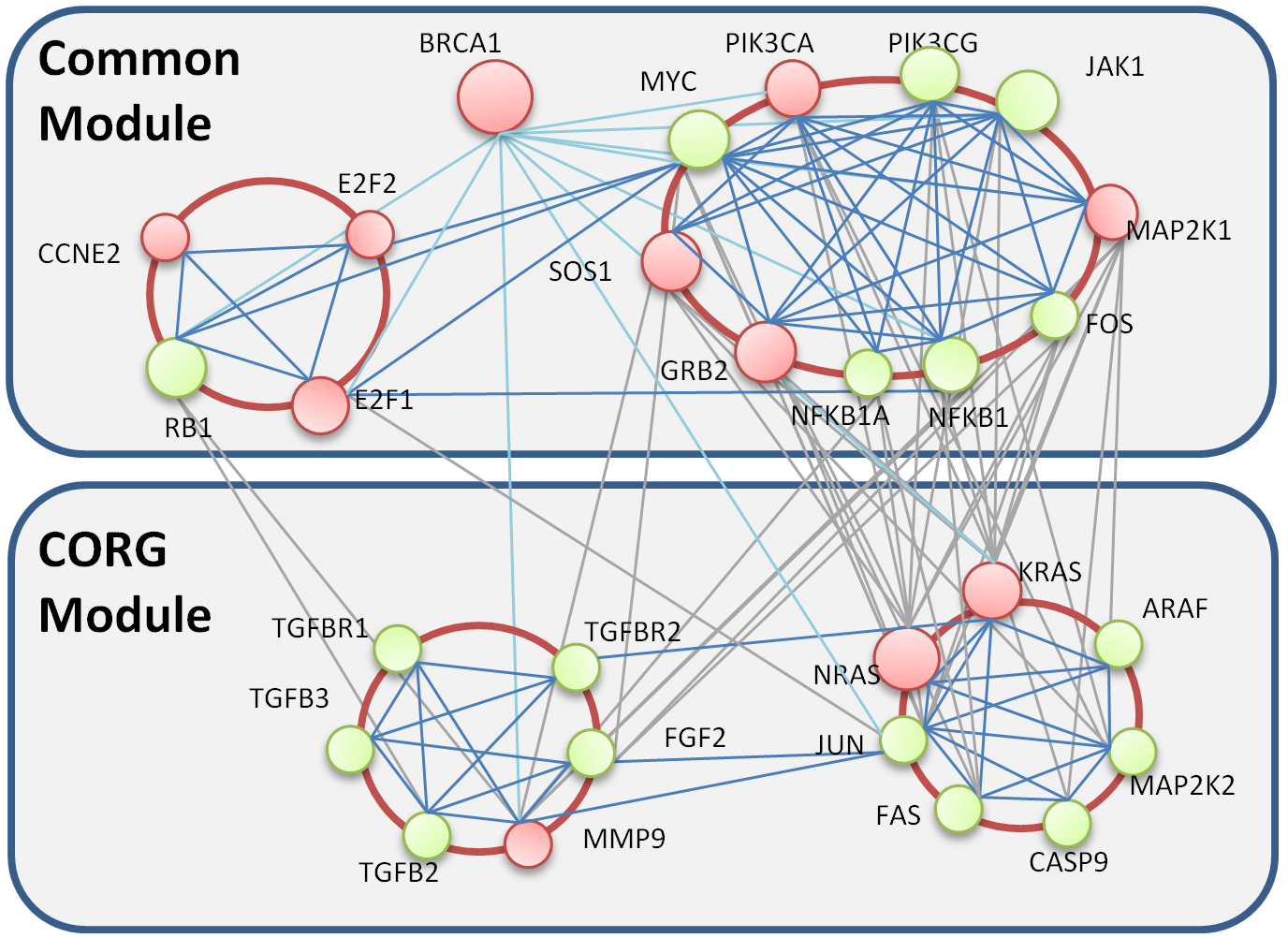

Supplement: Additional file 5 — Figure S2: Unique core module of cancer pathway identified by CORG-COMBINER method. [file 1471-2105-13-12-S5.TIFF]
